# Supplementary material for: On the effects of hard and soft equality constraints in the iterative outlier elimination procedure
Source: PLoS One. 2020 Aug 26;15(8):e0238145. doi: 10.1371/journal.pone.0238145 (PMC7449505; doi:10.1371/journal.pone.0238145)
Supplement: S1 Appendix — Provides a broad theoretical framework and detailed description of the method used to estimate the Iterative Data-Snooping probability levels. (PDF) [file pone.0238145.s001.pdf]

Supporting Information

Vinicius Francisco Rofatto[1,2*], Marcelo Tomio Matsuoka[1,2,3,6], Ivandro Klein[4,5], Maurício Roberto Veronez[6], Luiz Gonzaga da Silveira Junior[6]

1 Graduate Program in Remote Sensing, Federal University of Rio Grande do Sul, Porto Alegre, RS, Brazil
2 Institute of Geography, Federal University of Uberlândia, Monte Carmelo, MG, Brazil
3 Graduate Program in Agriculture and Geospatial Information, Federal University of Uberlândia, Monte Carmelo, MG, Brazil
4 Department of Civil Construction, Federal Institute of Santa Catarina, Florianópolis, SC, Brazil
5 Graduate Program in Geodetic Sciences, Federal University of Paraná, Curitiba, PR, Brazil
6 Graduate Program in Applied Computing, Unisinos University, São Leopoldo, RS, Brazil

* vfrofatto@gmail.com

1 Theoretical Framework

The *IDS* is an important case of multiple hypothesis testing. In this section, therefore, we briefly present the principal elements involved for the case of multiple testing.

First, the null hypothesis, denoted by $\mathcal{H}_0$, is formulated under the condition that random errors are normally distributed with expectation zero, i.e. in the absence of outliers. Thus, the null hypothesis $\mathcal{H}_0$ of the standard Gauss–Markov model in the linear or linearised form is given by Koch1999:

$$\mathcal{H}_0 : \mathbb{E}\{\boldsymbol{y}\} = \boldsymbol{A}\boldsymbol{x} + \mathbb{E}\{\boldsymbol{e}\} = \boldsymbol{A}\boldsymbol{x};\ \mathbb{D}\{\boldsymbol{y}\} = \boldsymbol{Q_e} \tag{1}$$

where $\mathbb{E}\{.\}$ is the expectation operator, $\mathbb{D}\{.\}$ is the dispersion operator, $\boldsymbol{y} \in \mathbb{R}^{n \times 1}$ is the vector of measurements, $\boldsymbol{A} \in \mathbb{R}^{n \times u}$ is the coefficient matrix, $\boldsymbol{x} \in \mathbb{R}^{u \times 1}$ is the unknown parameter vector, $\boldsymbol{e} \in \mathbb{R}^{n \times 1}$ is the unknown vector of measurement errors and $\boldsymbol{Q_e} \in \mathbb{R}^{n \times n}$ is the positive-definite covariance matrix of the measurements $\boldsymbol{y}$.

Under normal working conditions (i.e., $\mathcal{H}_0$), the measurement error model is then given by

$$\boldsymbol{e} \sim \boldsymbol{N}(\boldsymbol{0}, \boldsymbol{Q_e}), \tag{2}$$

First, we assume that the coefficient matrix $\boldsymbol{A}$ suffers from a rank deficiency, i.e. $u - rank(\boldsymbol{A}) > 0$. In that case, minimal constraints are added to solve the problem of the rank-deficient system. An example of how to handle the problem of the rank-deficient was given in the section **Problem description**, where a minimal constraint has been added to the matrix $\boldsymbol{A}$. This means that the columns of the design matrix $\boldsymbol{A}$ in Equation 1 become linearly independent, i.e. the matrix $\boldsymbol{A}$ become full rank, such that $u - rank(\boldsymbol{A}) = 0$.

The best linear unbiased estimator (BLUE) of $\boldsymbol{e}$ under $\mathcal{H}_0$ is the well-known estimated least-squares residual vector $\hat{\boldsymbol{e}} \in \mathbb{R}^{n \times 1}$, which is given by

$$
\begin{aligned}
\hat{\boldsymbol{e}} &= \boldsymbol{y} - \boldsymbol{A}\hat{\boldsymbol{x}} \\
&= \boldsymbol{y} - \boldsymbol{A}(\boldsymbol{A}^T\boldsymbol{W}\boldsymbol{A})^{-1}(\boldsymbol{A}^T\boldsymbol{W}\boldsymbol{y}) \\
&= \boldsymbol{A}\boldsymbol{x} + \boldsymbol{e} - \boldsymbol{A}(\boldsymbol{A}^T\boldsymbol{W}\boldsymbol{A})^{-1}(\boldsymbol{A}^T\boldsymbol{W}(\boldsymbol{A}\boldsymbol{x}+\boldsymbol{e})) \\
&= \boldsymbol{e} - \boldsymbol{A}(\boldsymbol{A}^T\boldsymbol{W}\boldsymbol{A})^{-1}(\boldsymbol{A}^T\boldsymbol{W}\boldsymbol{e}) \\
&= (\boldsymbol{I} - \boldsymbol{A}(\boldsymbol{A}^T\boldsymbol{W}\boldsymbol{A})^{-1}\boldsymbol{A}^T\boldsymbol{W})\boldsymbol{e} \\
&= \boldsymbol{R}\boldsymbol{e},
\end{aligned} \tag{3}
$$

with $\hat{\boldsymbol{x}} \in \mathbb{R}^{u\times 1}$ being the BLUE of $\boldsymbol{x}$ under $\mathcal{H}_0$; $\boldsymbol{W} \in \mathbb{R}^{n\times n}$ is the known matrix of weights, taken as $\boldsymbol{W} = {\sigma_0}^2\boldsymbol{Q}_{\boldsymbol{e}}^{-1}$, where σ_0^2 is the variance factor, $\boldsymbol{I} \in \mathbb{R}^{n\times n}$ is the identity matrix and $\boldsymbol{R} \in \mathbb{R}^{n\times n}$ is known as the redundancy matrix. The $\boldsymbol{R}$ matrix is an orthogonal projector that projects onto the orthogonal complement of the range space of $\boldsymbol{A}$. The main diagonal elements of matrix $\boldsymbol{R}$ are known as local redundancy numbers (denoted by r_i of the the system. The larger the number of local redundancy for a given measurement, the larger the degree of importance of that measurement for the model, i.e. the larger the absorption of a possible error of that measurement into their corresponding least-squares residual.

The degrees of freedom r (i.e. the overall redundancy) of the model under $\mathcal{H}_0$ (Equation (1)) is

$$
r = rank(\boldsymbol{Q}_{\hat{\boldsymbol{e}}}) = n - rank(\boldsymbol{A}) = n - u, where \tag{4}
$$

$$
\boldsymbol{Q}_{\hat{\boldsymbol{e}}} = \boldsymbol{Q}_{\boldsymbol{e}} - {\sigma_0}^2\boldsymbol{A}(\boldsymbol{A}^T\boldsymbol{W}\boldsymbol{A})^{-1}\boldsymbol{A}^T \tag{5}
$$

On the other hand, an alternative model is proposed when there are doubts about the reliability level of the model under $\mathcal{H}_0$. Here, we assume that the validity of the null hypothesis $\mathcal{H}_0$ in Equation (1) can be violated if the dataset is contaminated by outliers. The model in an alternative hypothesis, denoted by $\mathcal{H}_A$, is to oppose Equation (1) by an extended model that includes the unknown vector $\boldsymbol{\nabla} \in \mathbb{R}^{q\times 1}$ of deterministic bias parameters as follows ([1, 2]):

$$
\mathcal{H}_A : \boldsymbol{y} = \boldsymbol{A}\boldsymbol{x} + \boldsymbol{C}\boldsymbol{\nabla} + \boldsymbol{e}
$$

$$
= \begin{pmatrix}\boldsymbol{A} & \boldsymbol{C}\end{pmatrix}\begin{pmatrix}\boldsymbol{x}\\ \boldsymbol{\nabla}\end{pmatrix} + \boldsymbol{e}, \ (6)
$$

where $\boldsymbol{C} \in \mathbb{R}^{n\times q}$ is the matrix that relates bias parameters, i.e., the values of the outliers to observations. We restrict ourselves to the matrix $(\boldsymbol{A} \quad \boldsymbol{C})$ having full column rank, such that

$$
r = rank\begin{pmatrix}\boldsymbol{A} & \boldsymbol{C}\end{pmatrix} = u + q \leq n \tag{7}
$$

One of the most used procedures based on hypothesis testing for outliers in linear (or linearised) models is the well-known data snooping method [1, 3]. This procedure consists of screening each individual measurement for the presence of an outlier [4]. In that case, data snooping is based on a local model test, such that $q = 1$, and therefore, the n alternative hypothesis is expressed as

$$
\mathcal{H}_A^{(i)} : \boldsymbol{y} = \boldsymbol{A}\boldsymbol{x} + \boldsymbol{c_i}\nabla_i + \boldsymbol{e}
$$

$$
= \begin{pmatrix}\boldsymbol{A} & \boldsymbol{c_i}\end{pmatrix}\begin{pmatrix}\boldsymbol{x}\\ \nabla_i\end{pmatrix} + \boldsymbol{e}, \ \forall i = 1, \cdots, n(8)
$$

Now, matrix $\boldsymbol{C}$ in Equation (1) is reduced to a canonical unit vector $\boldsymbol{c_i}$, which consists exclusively of elements with values of 0 and 1, where 1 means that the ith bias

parameter of magnitude ∇_i affects the ith measurement, and 0 means otherwise. In that case, the rank of $(\boldsymbol{A} \quad \boldsymbol{c_i}) \in \mathbb{R}^{n\times(u+1)}$ and the vector $\boldsymbol{\nabla}$ in Equation (1) reduces to a scalar ∇_i in Equation (1), i.e., $\boldsymbol{c_i}{=}\begin{pmatrix}0 & 0 & 0 & \cdots & 1^{ith} & 0 & \cdots & 0\end{pmatrix}^T$. When $q = n - u$, an overall model test is performed. For more details about the overall model test, see, for example, [5, 6].

Note that the alternative hypothesis $\mathcal{H}_A^{(i)}$ in Equation (1) is formulated under the condition that the outlier acts as a systematic effect by shifting the random error distribution under $\mathcal{H}_0$ by its own value [7]. In other words, the presence of an outlier in a dataset can cause a shift of the expectation under $\mathcal{H}_0$ to a nonzero value. Therefore, hypothesis testing is often employed to check whether the possible shifting of the random error distribution under $\mathcal{H}_0$ by an outlier is, in fact, a systematic effect (bias) or merely a random effect. This hypothesis test-based approach is called the *mean-shift model* (see, e.g., [1, 3, 8–21].

In the context of the mean-shift model, the test statistic involved in data snooping is given by the normalised least-squares residual, denoted by $\boldsymbol{w_i}$. This test statistic, also known as Baarda's w-test, is given as follows:

$$\boldsymbol{w_i} = \frac{\boldsymbol{c_i}^T \boldsymbol{Q_e}^{-1} \hat{\boldsymbol{e}}}{\sqrt{\boldsymbol{c_i}^T \boldsymbol{Q_e}^{-1} \boldsymbol{Q_{\hat{e}}} \boldsymbol{Q_e}^{-1} \boldsymbol{c_i}}}, \forall i = 1, \cdots, n \tag{9}$$

The alternative hypothesis in Equation (1) is formulated in the sense that "*There is at least one outlier in the vector of measurements y_i*" [10]. In that case, we are interested in knowing which of the alternative hypotheses may lead to the rejection of the null hypothesis with a certain probability. This means testing $\mathcal{H}_0$ against $\mathcal{H}_A^{(1)}, \mathcal{H}_A^{(2)}, \mathcal{H}_A^{(3)}, \ldots, \mathcal{H}_A^{(n)}$. This is known as multiple hypothesis testing (see, e.g., [5, 8, 9, 17, 22–28]). In that case, the test statistic coming into effect is the maximum absolute Baarda's w-test value (denoted by max-w), which is computed as [17]

$$\text{max-}w = \max_{i\in\{1,\cdots,n\}} |\boldsymbol{w_i}| \tag{10}$$

The decision rule for this case is given by

$$\begin{aligned} & \quad\quad\quad\quad \textit{Accept} \;\; \mathcal{H}_0 \;\; \textit{if} \;\; \text{max-}w \le \hat{k} \\ & \textit{Otherwise}, \\ & \quad\quad\quad\quad \textit{Accept} \;\; \mathcal{H}_A^{(i)} \;\; \textit{if} \;\; \text{max-}w > \hat{k} \end{aligned} \tag{11}$$

The decision rule in Equation 11 says that if none of the n w-tests get rejected, then we accept the null hypothesis $\mathcal{H}_0$. If the null hypothesis $\mathcal{H}_0$ is rejected in any of the n tests, then one can only assume that detection occurred. In other words, if the max-w is larger than some percentile of its probability distribution (i.e., some critical value $\hat{k}$), then there is evidence that there is an outlier in the dataset. Therefore, "outlier detection" only informs us whether the null hypothesis $\mathcal{H}_0$ is accepted or not [29].

However, the detection does not tell us which alternative hypothesis $\mathcal{H}_A^{(i)}$ would have led to the rejection of the null hypothesis $\mathcal{H}_0$. The localisation of the alternative hypothesis, which would have rejected the null hypothesis, is a problem of "outlier identification". Outlier identification implies the execution of a search among the measurements for the most likely outlier. In other words, one seeks to find which of Baarda's w-test is the maximum absolute value max-w and if that max-w is greater than some critical value $\hat{k}$.

Therefore, the data snooping procedure of screening measurements for possible outliers is actually an important case of multiple hypothesis testing and not single

hypothesis testing. Moreover, note that outlier identification only happens when outlier detection necessarily exists; i.e., "outlier identification" only occurs when the null hypothesis $\mathcal{H}_0$ is rejected. However, correct detection does not necessarily imply correct identification [5, 17, 25].

In a special case of having only one single alternative hypothesis, one should decide between the null hypothesis $\mathcal{H}_0$ and only one single alternative hypothesis $\mathcal{H}_A^{(i)}$ of Equation (1). In that case, the false decisions are restricted to Type I error and Type II error. The probability of a Type I Error α_0 is the probability of rejecting the null hypothesis $\mathcal{H}_0$ when it is true, whereas the probability of a Type II error β_0 is the probability of failing to reject the null hypothesis $\mathcal{H}_0$ when it is false (note: the index '0' represents the case in which a single hypothesis is tested). Instead of α_0 and β_0, there is the confidence level $CL = 1 - \alpha_0$ and the power of the test $\gamma_0 = 1 - \beta_0$, respectively. The first deals with the probability of accepting a true null hypothesis $\mathcal{H}_0$; the second addresses the probability of correctly accepting the alternative hypothesis $\mathcal{H}_A^{(i)}$. In that case, given a probability of a Type I decision error α_0, we find the critical value k_0 as follows:

$$k_0 = \Phi^{-1}\left(1 - \frac{\alpha_0}{2}\right) \tag{12}$$

where Φ^{-1} denotes the inverse of the cumulative distribution function (cdf) of the two-tailed standard normal distribution $\boldsymbol{N}(0, 1)$.

The normalised least-squares residual $w_{\boldsymbol{i}}$ follows a standard normal distribution with the expectation that $\mathbb{E}\{w_{\boldsymbol{i}}\} = 0$ if $\mathcal{H}_0$ holds true (there is no outlier). On the other hand, if the system is contaminated with a single outlier at the ith location of the dataset (i.e., under $\mathcal{H}_A^{(i)}$), then the expectation of $w_{\boldsymbol{i}}$ is

$$\mathbb{E}\{w_{\boldsymbol{i}}\} = \sqrt{\lambda_0} = \sqrt{\boldsymbol{c_i}^T \boldsymbol{Q_e}^{-1} \boldsymbol{Q_{\hat{e}}} \boldsymbol{Q_e}^{-1} \boldsymbol{c_i} \nabla_i^2} \tag{13}$$

where λ_0 is the non-centrality parameter for $q = 1$. Note, therefore, that there is an outlier that causes the expectation of $w_{\boldsymbol{i}}$ to become $\sqrt{\lambda_0}$. The square-root of the non-centrality parameter $\sqrt{\lambda_0}$ in Equation (13) represents the expected mean shift of a specific w-test. In such a case, the term $\boldsymbol{c_i}^T \boldsymbol{Q_e}^{-1} \boldsymbol{Q_{\hat{e}}} \boldsymbol{Q_e}^{-1} \boldsymbol{c_i}$ in Equation (13) is a scalar, and therefore, it can be rewritten as follows [30]:

$$|\nabla_i| = MDB_{0_{(i)}} = \sqrt{\frac{\lambda_0}{\boldsymbol{c_i}^T \boldsymbol{Q_e}^{-1} \boldsymbol{Q_{\hat{e}}} \boldsymbol{Q_e}^{-1} \boldsymbol{c_i}}},\ \forall i = 1, \cdots, n \tag{14}$$

where $|\nabla_i|$ is the Minimal Detectable Bias ($MDB_{0_{(i)}}$) for the case in which there is only one single alternative hypothesis, which can be computed for each individual alternative hypothesis according to Equation (1).

For a single outlier, the variance of an estimated outlier, denoted by $\sigma^2_{\nabla_i}$, is

$$\sigma^2_{\nabla_i} = \left(\boldsymbol{c_i}^T \boldsymbol{Q_e}^{-1} \boldsymbol{Q_{\hat{e}}} \boldsymbol{Q_e}^{-1} \boldsymbol{c_i}\right)^{-1},\ \forall i = 1, \cdots, n \tag{15}$$

Thus, the MDB can also be written as

$$MDB_{0_{(i)}} = \sigma_{\nabla_i} \sqrt{\lambda_0},\ \forall i = 1, \cdots, n \tag{16}$$

where $\sigma_{\nabla_i} = \sqrt{\sigma^2_{\nabla_i}}$ is the standard deviation of estimated outlier ∇_i.

The MDB in Equations (14) or (16) of an alternative hypothesis is the smallest-magnitude outlier that can lead to the rejection of the null hypothesis $\mathcal{H}_0$ for a given α_0 and β_0. Thus, for each model of the alternative hypothesis $\mathcal{H}_A^{(i)}$, the corresponding MDB can be computed [17, 31, 32]. The limitation of this MDB is that it

was initially developed for the binary hypothesis testing case. In that case, the MDB is a sensitivity indicator of Baarda's w-test when only one single alternative hypothesis is taken into account. In this article, we are confined to multiple alternative hypotheses. Therefore, both the MDB and MIB are computed by considering the case of multiple hypothesis testing.

For a scenario coinciding with the null hypothesis $\mathcal{H}_0$ under multiple testing hypothesis, there is the probability of incorrectly identifying at least one alternative hypothesis. This type of wrong decision is known as the *family-wise error rate* (FWE). The FWE is defined as

$$FWE = \alpha' =\leq 1 - (1 - \alpha_0)^n \tag{17}$$

which is approximately

$$FWE = \alpha' \leq n \times \alpha_0 \tag{18}$$

where α_0 is the significance level for an individual test. The quantity in Equation (18) is just equal to the upper bound of the Bonferroni inequality, i.e., $\alpha' \leq n\alpha$ [33]. For example, if the FWE level (α') is 0.05 and one is running 5 tests, then each test will have an α_0 of $0.05/5 = 0.01$. In other words, one uses a global Type I Error rate α' that combines all tests under consideration instead of an individual error rate α_0 that only considers one test at a time time [27]. In that case, the critical value k_{bonf} is computed as

$$k_{bonf} = \Phi^{-1}\left(1 - \frac{\alpha'}{2n}\right) \tag{19}$$

The Bonferroni in Equation (18) is a good approximation for the case in which alternative hypotheses are independent. In practice, however, the test results always depend on each other to some degree because we always have a correlation between w-tests. The correlation coefficient between any Baarda's w-test statistic (denoted by $\rho_{\boldsymbol{w_i},\boldsymbol{w_j}}$), such as $\boldsymbol{w_i}$ and $\boldsymbol{w_j}$, is given by [8]

$$\rho_{\boldsymbol{w_i},\boldsymbol{w_j}} = \frac{\boldsymbol{c_i}^T\boldsymbol{Q_e}^{-1}\boldsymbol{Q_{\hat{e}}}\boldsymbol{Q_e}^{-1}\boldsymbol{c_j}}{\sqrt{\boldsymbol{c_i}^T\boldsymbol{Q_e}^{-1}\boldsymbol{Q_{\hat{e}}}\boldsymbol{Q_e}^{-1}\boldsymbol{c_i}}\sqrt{\boldsymbol{c_j}^T\boldsymbol{Q_e}^{-1}\boldsymbol{Q_{\hat{e}}}\boldsymbol{Q_e}^{-1}\boldsymbol{c_j}}}, \forall(i \neq j) \tag{20}$$

The correlation coefficient $\rho_{\boldsymbol{w_i},\boldsymbol{w_j}}$ can assume values within the range $[-1, 1]$.

Here, the extreme normalised residuals max-w (i.e., maximum absolute) in Equation (10) are treated directly as a test statistic. Note that when using Equation (10) as a test statistic, the decision rule is based on a one-sided test of the form max-$w \leq \hat{k}$. However, the distributions of max-w cannot be derived from well-known test distributions (e.g., normal distribution). The procedure to compute the critical value of max-w is given step-by-step by Rofatto et al. [29].

The other side of the multiple testing problem is the situation in which there is an outlier in the dataset. In that case, apart from Type I and Type II errors, there is a third type of wrong decision associated with Baarda's w-test. Baarda's w-test can also flag a non-outlying observation while the '*true*' outlier remains in the dataset. We are referring to the Type III error [23], also referred to as the probability of wrong identification ($\mathcal{P}_{WI}$). The description of the Type III error involves a separability analysis between alternative hypotheses [8, 22, 25, 26]. Therefore, we are now interested in the identification of the correct alternative hypothesis. In that case, the non-centrality parameter in Equation (13) is not only related to the sizes of Type I and Type II decision errors but also dependent on the correlation coefficient $\rho_{\boldsymbol{w_i},\boldsymbol{w_j}}$ given by Equation (20).

On the basis of the assumption that one outlier is in the ith position of the dataset (i.e., $\mathcal{H}_A^{(i)}$ is '*true*'), the probability of a Type II error (also referenced as the probability

of "*missed detection*", denoted by $\mathcal{P}_{MD}$) for multiple testing is

$$\mathcal{P}_{MD} = \mathcal{P}\left(\bigcap_{i=1}^{n} |\boldsymbol{w_i}| \leq \hat{k} \;\middle|\; \mathcal{H}_A^{(i)} : true\right), \tag{21}$$

and the size of a Type III wrong decision (also called "*misidentification*", denoted by $\mathcal{P}_{WI}$) is given by

$$\mathcal{P}_{WI} = \sum_{i=1}^{n} \mathcal{P}\left(|\boldsymbol{w_j}| > |\boldsymbol{w_i}| \;\forall i,\; |\boldsymbol{w_j}| > \hat{k}(i \neq j) \;\middle|\; \mathcal{H}_A^{(i)} : true\right) \tag{22}$$

On the other hand, the probability of correct identification (denoted by $\mathcal{P}_{CI}$) is

$$\mathcal{P}_{CI} = \mathcal{P}\left(|\boldsymbol{w_i}| > |\boldsymbol{w_j}| \;\forall j,\; |\boldsymbol{w_i}| > \hat{k}(i \neq j) \;\middle|\; \mathcal{H}_A^{(i)} : true\right) \tag{23}$$

with

$$1 - \mathcal{P}_{CI} = 1 - \mathcal{P}_{CD} + \mathcal{P}_{WI} = \mathcal{P}_{MD} + \mathcal{P}_{WI} \tag{24}$$

Note that the three probabilities of missed detection $\mathcal{P}_{MD}$, wrong identification $\mathcal{P}_{WI}$ and correct identification $\mathcal{P}_{CI}$ sum up to unity: i.e., $\mathcal{P}_{MD} + \mathcal{P}_{WI} + \mathcal{P}_{CI} = 1$.

The probability of correct detection $\mathcal{P}_{CD}$ is the sum of the probability of correct identification $\mathcal{P}_{CI}$ (selecting a correct alternative hypothesis) and the probability of misidentification $\mathcal{P}_{WI}$ (selecting one of the n-1 other hypotheses), i.e.,

$$\mathcal{P}_{CD} = \mathcal{P}_{CI} + \mathcal{P}_{WI} \tag{25}$$

The probability of wrong identification $\mathcal{P}_{WI}$ is identically zero, $\mathcal{P}_{WI} = 0$, when the correlation coefficient is exactly zero, $\rho_{\boldsymbol{w_i},\boldsymbol{w_j}} = 0$. In that case, we have

$$\mathcal{P}_{CD} = \mathcal{P}_{CI} = 1 - \mathcal{P}_{MD} \tag{26}$$

The relationship given in Equation (26) would only happen if one neglected the nature of the dependence between alternative hypotheses. In other words, this relationship is valid for the special case of testing the null hypothesis $\mathcal{H}_0$ against only one single alternative hypothesis $\mathcal{H}_A^{(i)}$.

Since the critical region in multiple hypothesis testing is larger than that in single hypothesis testing, the Type II decision error (i.e., $\mathcal{P}_{MD}$) for the multiple test becomes smaller [17]. This means that the correct detection in binary hypothesis testing (γ_0) is smaller than the correct detection $\mathcal{P}_{CD}$ under multiple hypothesis testing, i.e.,

$$\mathcal{P}_{CD} > \gamma_0 \tag{27}$$

Detection is easier in the case of multiple hypothesis testing than single hypothesis testing. However, the probability of correct detection $\mathcal{P}_{CD}$ under multiple testing is spread out over all alternative hypotheses, and therefore, identifying is harder than detecting. From Equation (25), it is also noted that detection does not depend on identification. However, outlier identification depends on correct outlier detection. Therefore, we have the following inequality:

$$\mathcal{P}_{CI} \leq \mathcal{P}_{CD} \tag{28}$$

Note that the probability of correct identification $\mathcal{P}_{CI}$ depends on the probability of missed detection $\mathcal{P}_{MD}$ and wrong identification $\mathcal{P}_{WI}$ for the case in which data snooping is run only once, i.e., a single round of estimation and testing. However, in this paper, we deal with data snooping in its iterative form (i.e., *IDS*), and therefore, the probability of correct identification $\mathcal{P}_{CI}$ depends on other decision rules.

In contrast to the data snooping single run, the success rate of correct detection $\mathcal{P}_{CD}$ for *IDS* depends on the sum of the probabilities of correct identification $\mathcal{P}_{CI}$, wrong exclusion ($\mathcal{P}_{WE}$), over-identification cases ($\mathcal{P}_{over+}$ and $\mathcal{P}_{over-}$), and statistical overlap ($\mathcal{P}_{ol}$), i.e.,

$$\mathcal{P}_{CD} = 1 - \mathcal{P}_{MD} = \mathcal{P}_{CI} + \mathcal{P}_{WE} + \mathcal{P}_{over+} + \mathcal{P}_{over-} + \mathcal{P}_{ol} \tag{29}$$

It is important to mention that the probability of correct detection is the complement of the probability of missed detection. Note from Equation (29) that the probability of correct detection $\mathcal{P}_{CD}$ is available even for cases in which the identification rate is null, $\mathcal{P}_{CI} = 0$. However, the probability of correct identification ($\mathcal{P}_{CI}$) necessarily requires that the probability of correct detection $\mathcal{P}_{CD}$ be greater than zero. For the same reasons given for the data snooping single run in the previous section, detecting is easier than identifying. In that case, we have the following relationship for the success rate of correct outlier identification $\mathcal{P}_{CI}$:

$$\mathcal{P}_{CI} = \mathcal{P}_{CD} - (\mathcal{P}_{WE} + \mathcal{P}_{over+} + \mathcal{P}_{over-} + \mathcal{P}_{ol}), \tag{30}$$

such as

$$\exists(\mathcal{P}_{CI}) \in [0,1] \iff (\mathcal{P}_{CD}) > 0 \tag{31}$$

It is important to mention that the wrong exclusion $\mathcal{P}_{WE}$ describes the probability of identifying and removing a non-outlying measurement while the '*true*' outlier remains in the dataset. In other words, $\mathcal{P}_{WE}$ is the Type III decision error for *IDS*). The overall wrong exclusion $\mathcal{P}_{WE}$ is the result of the sum of each individual contribution to $\mathcal{P}_{WE}$, i.e.,

$$\mathcal{P}_{WE} = \sum_{i=1}^{n-1} \mathcal{P}_{WE(i)} \tag{32}$$

On the basis of the probability levels of correct detection $\mathcal{P}_{CD}$ and correct identification $\mathcal{P}_{CI}$, the sensitivity indicators of minimal biases—Minimal Detectable Bias (MDB) and Minimal Identifiable Bias (MIB)—for a given α' can be computed as follows:

$$MDB = \arg\min_{\nabla_i} \mathcal{P}_{CD}(\nabla_i) > \tilde{\mathcal{P}}_{CD}, \forall i = 1, \cdots, n \tag{33}$$

$$MIB = \arg\min_{\nabla_i} \mathcal{P}_{CI}(\nabla_i) > \tilde{\mathcal{P}}_{CI}, \forall i = 1, \cdots, n \tag{34}$$

Equation (33) gives the smallest outlier ∇_i that leads to its detection for a user-defined correct detection rate $\tilde{\mathcal{P}}_{CD}$, whereas (34) provides the smallest outlier ∇_i that leads to its identification for a user-defined correct identification rate $\tilde{\mathcal{P}}_{CI}$.

As a consequence of the inequality in (28), the MIB will be larger than MDB, i.e., $MIB \geq MDB$. For the special case of having only one single alternative hypothesis, there is no difference between the MDB and MIB [5]. The computation of MDB_0 is easily performed by Equations (14) or (16), whereas the computation of the MDB in Equation (33) and the MIB in Equation (34) must be computed using Monte Carlo because the acceptance region (as well as the critical region) for the case of multiple alternative hypotheses is analytically intractable.

2 Description of the Method

The procedure to compute the critical value of max-w ($\hat{k}$) is given step-by-step as follows:

1. Specify the probability density function (pdf) of the w-test statistics. The pdf assigned to the w-test statistics under an $\mathcal{H}_0$-distribution is

$$(w_1, w_2, w_3, \cdots, w_n)^T \sim N(\mathbf{0}, \boldsymbol{\mathcal{R}}_{\boldsymbol{w}}) \tag{35}$$

where $\boldsymbol{\mathcal{R}}_{\boldsymbol{w}} \in \mathbb{R}^{n \times n}$ is the correlation matrix with the main diagonal elements equal to 1, and the off-diagonal elements are the correlation between the w-test statistics computed by Equation (20).

2. In order to have w-test statistics under $\mathcal{H}_0$, uniformly distributed random number sequences are produced by the Mersenne Twister algorithm, and then they are transformed into a normal distribution by using the Box–Muller transformation [34]. Box–Muller has already been used in geodesy for Monte Carlo experiments [10, 35, 36]. Therefore, a sequence of m random vectors from the pdf assigned to the w-test statistics is generated according to Equation (35). In that case, we have a sequence of m vectors of the w-test statistics as follows:

$$\left[(w_1, w_2, w_3, \cdots, w_n)^{T^{(1)}}, (w_1, w_2, w_3, \cdots, w_n)^{T^{(2)}}, \cdots, (w_1, w_2, w_3, \cdots, w_n)^{T^{(m)}}\right] \tag{36}$$

3. Compute the test statistic by Equation (10) for each sequence of w-test statistics. Thus, we have

$$\left(\max_{i \in \{1, \cdots, n\}} |\boldsymbol{w_i}|^{(1)}, \max_{i \in \{1, \cdots, n\}} |\boldsymbol{w_i}|^{(2)}, \cdots, \max_{i \in \{1, \cdots, n\}} |\boldsymbol{w_i}|^{(m)}\right) \tag{37}$$

4. Sort in ascending order the maximum test statistic in Equation (37), getting a sorted vector $\tilde{w}$, such that

$$\tilde{w}^{(1)} < \tilde{w}^{(2)}, \tilde{w}^{(3)}, \cdots, < \tilde{w}^{(m)} \tag{38}$$

The sorted values $\tilde{w}$ in Equation (38) provide a discrete representation of the cumulative density function (cdf) of the maximum test statistic max-w.

5. Determine the critical value $\hat{k}$ as follows:

$$\hat{k} = \tilde{w}_{[(1-\alpha') \times m]} \tag{39}$$

where [.] denotes rounding down to the next integer that indicates the position of the selected elements in the ascending order of $\tilde{w}$. This position corresponds to a critical value for a stipulated overall false alarm probability α'. This can be done for a sequence of values α' in parallel.

It is important to mention that the probability of a Type I decision error for multiple testing α' is larger than that of Type I for single testing α_0. This is because the critical region in multiple testing is larger than that in single hypothesis testing.

After finding the critical value $\hat{k}$, the procedure based on Monte Carlo is also applied to compute the probability levels of *IDS* when there is an outlier in the dataset. The overview of of the main elements involved with the *IDS* was detailed in previous section.

The steps of the method are detailed as follows:

1. First, random error vectors are synthetically generated on the basis of a multivariate normal distribution because the assumed stochastic model for random errors is based on the matrix covariance of the observations. Here, we use the Mersenne Twister algorithm [37] to generate a sequence of random numbers and Box–Muller [34] to transform it into a normal distribution.

2. The total error ($\boldsymbol{\varepsilon}$) is a combination of random errors, and its corresponding outlier is given as follows:

$$\boldsymbol{\varepsilon} = \boldsymbol{e} + \boldsymbol{c_i}\nabla_i \tag{40}$$

   The magnitude intervals of simulated outliers are user-defined. The magnitude intervals are based on the standard deviation of the observation (σ), e.g., $|3\sigma|$ to $|6\sigma|$. Since the outlier can be positive or negative, the proposed algorithm randomly selects the signal of the outlier (for $q = 1$). Here, we use the discrete uniform distribution to select the signal of the outlier.

   In Equation (40), $\boldsymbol{e}$ is the random error generated from the normal distribution according to Equation (2), and the second part $\boldsymbol{c_i}\nabla_i$ is the additional parameter that describes the alternative model according to Equation (40).

3. Next, we compute the least-squares residuals vector according to Equation (3), but now we use the total error ($\boldsymbol{\varepsilon}$) in Equation 40 as follows:

$$\hat{\boldsymbol{e}} = \boldsymbol{R}\boldsymbol{\varepsilon} \tag{41}$$

4. Run the *IDS*. In this step, the test statistic is computed according to (9). Then, the maximum test statistic value is obtained according to Equation (10). Now, the decision rule is based on the critical value $\hat{k}$ computed by Monte Carlo in previous stage. After identifying the measurement suspected to be the most likely outlier, it is excluded from the model, and least-squares estimation and data snooping are applied iteratively until there are no further outliers identified in the dataset. If two or more observations are simultaneously detected (i.e. if $max - w > \hat{k}$ and the size of $max - w > 1$, then the IDS is ended). Furthermore, every time that a measurement suspected to be the most likely outlier is removed from the model, we check whether the normal matrix $\boldsymbol{A}^T\boldsymbol{W}\boldsymbol{A}$ is invertible or not. If the determinant of $\boldsymbol{A}^T\boldsymbol{W}\boldsymbol{A}$ is 0, $det|\boldsymbol{A}^T\boldsymbol{W}\boldsymbol{A}| = 0$, then there is a necessary and sufficient condition for a square matrix $\boldsymbol{A}^T\boldsymbol{W}\boldsymbol{A}$ to be non-invertible. In other words, the *IDS* is ended when $det|\boldsymbol{A}^T\boldsymbol{W}\boldsymbol{A}| = 0$. If no outlier is detected (i.e. $max - w < \hat{k}$), then the IDS is also ended.

   The *IDS* procedure is performed for m experiments of random error vectors for each experiment contaminated by an outlier in the ith measurement. Therefore, for each measurement contaminated by an outlier, there are $v = 1, \ldots, m$ experiments.

5. After running m=200,000 experiments [17], the probabilities associated with *IDS* are computed (note: m refers to the total number of Monte Carlo experiments). The probability of correct identification ($\mathcal{P}_{CI}$) is the ratio between the number of times that the outlier is correctly identified (denoted as n_{CI}) and m experiments, i.e.:

$$\mathcal{P}_{CI} = \frac{n_{CI}}{m} \tag{42}$$

   Similar to Equation (42), the wrong decisions are computed as

$$\mathcal{P}_{MD} = \frac{n_{MD}}{m} \tag{43}$$

   where n_{MD} is the number of experiments in which *IDS* does not detect the outlier ($\mathcal{P}_{MD}$ corresponds to the rate of missed detection).

$$\mathcal{P}_{WE} = \frac{n_{WE}}{m} \tag{44}$$

where n_{WE} is the number of experiments in which the *IDS* procedure flags and removes only one single non-outlying measurement while the '*true*' outlier remains in the dataset ($\mathcal{P}_{WE}$ is the wrong exclusion rate).

$$\mathcal{P}_{over+} = \frac{n_{over_+}}{m} \tag{45}$$

where n_{over+} is the number of experiments in which *IDS* correctly identifies and removes the outlying measurement and others, and $\mathcal{P}_{over+}$ corresponds to its probability.

$$\mathcal{P}_{over-} = \frac{n_{over_-}}{m} \tag{46}$$

where n_{over-} represents the number of experiments in which *IDS* identifies and removes more than one non-outlying measurement, whereas the '*true outlier*' remains in the dataset ($\mathcal{P}_{over-}$ is the probability corresponding to this error probability class).

$$\mathcal{P}_{ol} = \frac{n_{ol}}{m} \tag{47}$$

where n_{ol} is the number of experiments in which the detector in Equation (10) flags two (or more) measurements simultaneously during a given iteration of *IDS*. Here, this is referred to as the number of statistical overlap n_{ol}, and $\mathcal{P}_{ol}$ corresponds to its probability.

6. Finally, the sensitivity indicators (MDB and MIB) are computed based on Equation 33 and Equation 34, respectively.

References

1. Baarda W. A testing procedure for use in geodetic networks. Publ on geodesy, New Series. 1968;2(5).

2. Teunissen PJG. Testing Theory: an introduction. 2nd ed. Delft University Press; 2006.

3. Baarda W. Statistical concepts in geodesy. Publ on geodesy, New Series. 1967;2(4).

4. Kok JJ, States U. On data snooping and multiple outlier testing [microform] / Johan J. Kok. U.S. Dept. of Commerce, National Oceanic and Atmospheric Administration, National Ocean Service, Charting and Geodetic Services : For sale by the National Geodetic Information Center, NOAA Rockville, Md; 1984.

5. Imparato D, Teunissen PJG, Tiberius CCJM. Minimal Detectable and Identifiable Biases for quality control. Surv Rev. 2019;51(367):289–299. doi:10.1080/00396265.2018.1437947.

6. Teunissen PJG. Distributional theory for the DIA method. Journal of Geodesy. 2018;92(1):59–80. doi:10.1007/s00190-017-1045-7.

7. Lehmann R. On the formulation of the alternative hypothesis for geodetic outlier detection. J Geod. 2013;87(4):373–386. doi:10.1007/s00190-012-0607-y.

8. Förstner W. Reliability and discernability of extended Gauss-Markov models. In: Seminar on mathematical models of Geodetic/Photogrammetric Point Determination with Regard to Outliers and Systematic Errors. vol. Series A. Deutsche Geodätische Kommision, Munich, Germany; 1983. p. 79–103.

9. Wang J, Knight NL. New Outlier Separability Test and Its Application in GNSS Positioning; 2012.

10. Lehmann R. Improved critical values for extreme normalized and studentized residuals in Gauss–Markov models. J Geod. 2012;86(12):1137–1146. doi:10.1007/s00190-012-0569-0.

11. Velsink H. On the deformation analysis of point fields. J Geod. 2015;89(11):1071–1087. doi:10.1007/s00190-015-0835-z.

12. Lehmann R, Lösler M. Multiple Outlier Detection: Hypothesis Tests versus Model Selection by Information Criteria. J Surv Eng. 2016;142(4):04016017. doi:10.1061/(ASCE)SU.1943-5428.0000189.

13. Teunissen PJG, Imparato D, Tiberius CCJM. Does RAIM with Correct Exclusion Produce Unbiased Positions? Sensors. 2017;17(7). doi:10.3390/s17071508.

14. Klein I, Matsuoka MT, Guzatto MP, Nievinski FG. An approach to identify multiple outliers based on sequential likelihood ratio tests. Surv Rev. 2017;49(357):449–457. doi:10.1080/00396265.2016.1212970.

15. Lehmann R, Lösler M. Congruence analysis of geodetic networks – hypothesis tests versus model selection by information criteria. J Appl Geod. 2017;11(4):271–283. doi:10.1515/jag-2016-0049.

16. Rofatto VF, Matsuoka MT, Klein I. DESIGN OF GEODETIC NETWORKS BASED ON OUTLIER IDENTIFICATION CRITERIA: AN EXAMPLE APPLIED TO THE LEVELING NETWORK. Bull Geod Sci. 2018;24(2):152–170.

17. Rofatto VF, Matsuoka MT, Klein I, Veronez MR, Bonimani ML, Lehmann R. A half-century of Baarda's concept of reliability: a review, new perspectives, and applications. Surv Rev. 2018;0(0):1–17. doi:10.1080/00396265.2018.1548118.

18. Nguyen VK, Renault [U+FFFD] Milocco R. Environment Monitoring for Anomaly Detection System Using Smartphones. Sensors. 2019;19(18). doi:10.3390/s19183834.

19. Nie Y, Yang L, Shen Y. Specific Direction-Based Outlier Detection Approach for GNSS Vector Networks. Sensors. 2019;19(8). doi:10.3390/s19081836.

20. Klein I, Matsuoka MT, Guzatto MP, Nievinski FG, Veronez MR, Rofatto VF. A new relationship between the quality criteria for geodetic networks. Journal of Geodesy. 2019;93(4):529–544. doi:10.1007/s00190-018-1181-8.

21. Matsuoka MT, Rofatto VF, Klein I, Roberto Veronez M, da Silveira LG, Neto JBS, et al. Control Points Selection Based on Maximum External Reliability for Designing Geodetic Networks. Applied Sciences. 2020;10(2). doi:10.3390/app10020687.

22. Mierlo JV. Statistical Analysis of Geodetic Measurements for the Investigation of Crustal Movements. In: Whitten CA, Green R, Meade BK, editors. Recent Crustal Movements, 1977. vol. 13 of Developments in Geotectonics. Elsevier; 1979. p. 457 – 467. Available from: http://www.sciencedirect.com/science/article/pii/B9780444417831500726.

23. Hawkins DM. Identification of Outliers. 1st ed. Springer Netherlands; 1980.

24. van der Marel H, Rösters AJM. Statistical Testing and Quality Analysis in 3-D Networks (part II) Application to GPS. In: Bock Y, Leppard N, editors. Global Positioning System: An Overview. New York, NY: Springer New York; 1990. p. 290–297.

25. Yang L, Wang J, Knight NL, Shen Y. Outlier separability analysis with a multiple alternative hypotheses test. J Geod. 2013;87(6):591–604. doi:10.1007/s00190-013-0629-0.

26. Prószyński W. Revisiting Baarda's concept of minimal detectable bias with regard to outlier identifiability. J Geod. 2015;89(10):993–1003. doi:10.1007/s00190-015-0828-y.

27. Romano JP, Wolf M. Multiple Testing of One-Sided Hypotheses: Combining Bonferroni and the Bootstrap. In: Kreinovich V, Sriboonchitta S, Chakpitak N, editors. Predictive Econometrics and Big Data. Cham: Springer International Publishing; 2018. p. 78–94.

28. Zaminpardaz S, Teunissen PJG. DIA-datasnooping and identifiability. J Geod. 2019;93(1):85–101. doi:10.1007/s00190-018-1141-3.

29. Rofatto VF, Matsuoka MT, Klein I, Roberto Veronez M, da Silveira LG. A Monte Carlo-Based Outlier Diagnosis Method for Sensitivity Analysis. Remote Sensing. 2020;12(5). doi:10.3390/rs12050860.

30. Teunissen PJG. An Integrity and Quality Control Procedure for use in Multi Sensor Integration; 1990.

31. Aydin C, Demirel H. Computation of Baarda's lower bound of the non-centrality parameter. J Geod. 2004;78(7):437–441. doi:10.1007/s00190-004-0406-1.

32. Aydin C. Power of Global Test in Deformation Analysis. J Surv Eng. 2012;138(2):51–56.

33. Bonferroni CE. Teoria statistica delle classi e calcolo delle probabilità. Pubblicazioni del R. Istituto superiore di scienze economiche e commerciali di Firenze. Libreria internazionale Seeber; 1936. Available from: `https://books.google.com.br/books?id=3CY-HQAACAAJ`.

34. Box GEP, Muller ME. A Note on the Generation of Random Normal Deviates. The Annals of Mathematical Statistics. 1958;29(2):610–611.

35. Lemeshko BY, Lemeshko SB. Extending the Application of Grubbs-Type Tests in Rejecting Anomalous Measurements. Measurement Techniques. 2005;48(6):536–547. doi:10.1007/s11018-005-0179-9.

36. Lehmann R, Scheffler T. Monte Carlo based data snooping with application to a geodetic network. J Appl Geod. 2011;5(3-4):123–134.

37. Matsumoto M, Nishimura T. Mersenne twister: A 623-dimensionally equidistributed uniform pseudo-random number generator. ACM Transactions on Modeling and Computer Simulation. 1998;8(1):3–30.
