# Supplementary material for: On the effects of hard and soft equality constraints in the iterative outlier elimination procedure
Source: PLoS One. 2020 Aug 26;15(8):e0238145. doi: 10.1371/journal.pone.0238145 (PMC7449505; doi:10.1371/journal.pone.0238145)
Supplement: Attachment — Submitted filename: Response to Reviewers.pdf [file pone.0238145.s002.pdf]

July 15, 2020
To: Qichun 'Kit' Zhang,
Academic Editor, PLOS ONE

Manuscript Number: PONE-D-20-10931R1.
Title: "On the Effects of Hard and Soft Equality Constraints in the Iterative Outlier Elimination Procedure"

Dear Editor,

On behalf of my co-authors, thank you for your letter and the opportunity to review our paper for consideration by PLONE ONE. The authors would also like to thank the referees for their indispensable collaborations and suggestions to this work. The suggestions offered by the reviewers have been immensely helpful, and we also appreciate their comments and other aspects of the paper.

We have carefully reviewed the comments and thoroughly revised the manuscript accordingly. Our responses are given in a point-by-point manner below, indicating exactly how we addressed each concern or problem and describing the changes we have made.

We would like to update our Funding Statement, as follows:

- The CNPq - Conselho Nacional de Desenvolvimento Científico e Tecnológico - Brasil had the role of providing the study grant for the second author (proc. nº103587/2019-5);

- The PETROBRAS (Grant Number 2018/00545-0) had the role of paying both the publication fee and the professional language editing service.

We have provided the following files as requested:

- English language editing certificate by MDPI.
- A copy of our manuscript showing our changes by highlighting them.
- A clean copy of the edited manuscript.

We hope the revised version is now suitable for publication and look forward to hearing from you in due course.

Thank you again for your attention and consideration.

Sincerely yours,

Vinicius Francisco Rofatto
Professor
Federal University of Uberlandia
Surveying and Cartographic Engineering
Email: vfrofatto@gmail.com /vinicius.rofatto@ufu.br

Reviewer's Comments:

Reviewer #1:

1) *Improve the quality of Figure 1. Flowchart of the algorithm because the text inside the boxes is hard to read.*
   Response: Thank you for pointing out this issue. We improve the Figure 1 as requested.

2) *Page 3 / 20, Line 85, the words " the minimal biases, MDB (Minimal Detectable Bias) and MIB (Minimal Identifiable Bias) ", the same words defined again just with a different style as Page 4 / 20 line 108 " the minimal biases - Minimal Detectable Bias (MDB) and Minimal Identifiable Bias (MIB) ", Please use the same style throughout the paper and secondly once an abbreviation is defined just use the same if required. The same repeated on page 7 / 20 lines 227 MIB (Minimal Identifiable Bias).*

   Response: We correct accordingly.

3) *Page 6 / 20 line 168. The expression 1/0=∞ is not true. 1/0 is said to be undefined because the division is defined in terms of multiplication. a/b = x is defined to mean that b*x = a. There is no x such that 0*x = 1, since 0*x = 0 for all x. Thus 1/0 does not exist, or is not defined, or is undefined.*

   Response: We agree and corrected this statement in the text. Please see page 6/19, line 166.

4) *In Table 1 with two hard constraints, why the local redundancy, the standard deviation least square estimated outlier, and the maximum absolute correlation all are equal for twelve measurements. While for 1 hard constraint and 3 hard constraints there is some variation present in twelve measurements.*

   Response: This is due to the geometry of the network. For different network geometries, there are different local redundancy, standard deviation least square estimated outlier, and the maximum absolute correlation. Important to observe that the geometry of the network can promote the correlation between the least-squares residuals, even with independent observables.

5) *Page 9 / 20. The abbreviations used are very much common please stop this work from line 254 to 259, probabilities of correct identification (PCI) and correct detection (PCD) used and defined 3 and 4 times, respectively. The other abbreviations also repeated quite often i.e. over-identification cases (P over+ and P over-) and the statistical overlap (Pol).*

   Response: The adjustments were made according to the reviewer's observations.

6) *Check the value of MIB in Table 4, column 3 is it the value equal to 25? Check the value of MIB in Table 6, column 3 is it the value equal to 22?*

   Response: Thank you for these notes. Both 25σ and 22σ are correct, and refer to a MIB of 25 mm and 22 mm with 80% of probability, respectively.

7) *Page 12 / 20 line 324, Cluster 4: MDB e MIB were equal for all cases. Here the lower case e stands for.*

   Response: It was a typing error. Here "e" is actually "and".

8) *In last some special attention must be given to the language as well. There are some sentences which completely wrong.*
Response: The text has been checked for correct use of grammar and common technical terms, and edited to a level suitable for reporting research in a scholarly journal. A professional English review service was hired and carried out and the certificate sent to the editor.

Reviewer #2:

1) *Reduce the length of Conclusion section. Only include important findings.*

Response: Thank you for that observation. We highlight only the main findings of this research in the Conclusion section.

2) *Include high quality figures. Figures should be of same size.*

Response: The Preflight Analysis and Conversion Engine (PACE) digital diagnostic tool has been used to ensure that figures meet PLOS requirements. All figures were properly formatted according to PLOS specifications.

3) *There are some missing lines or extra legend items in Figures 4(c) - 4(h).*

Response: Similar results end up overlapping and, therefore, the lines were formatted with different types. Figure 4 (h), for example, shows dashed line (green) for the case of 1 hard constraint, solid line (blue) for the case of 2 hard constraints and dashed line (red) for the case of 3 hard constraints.
